# Transient Receptor Potential Channels in Cardiovascular and Renal Diseases

**DOI:** 10.3390/cells11243960

**Published:** 2022-12-07

**Authors:** Shuangtao Ma, Michael X. Zhu

**Affiliations:** 1 Division of Nanomedicine and Molecular Intervention, Department of Medicine, Michigan State University, East Lansing, MI 48824, USA; 2 Department of Integrative Biology and Pharmacology, McGovern Medical School, University of Texas Health Science Center at Houston, Houston, TX 77030, USA

Transient receptor potential (TRP) channels belong to a superfamily of integral membrane proteins with diverse functions in sensory perception and cellular physiology. TRP channels are found in a wide range of organisms, from yeast to mammals, and are expressed in both excitable and non-excitable cells. A growing body of evidence indicates that TRP channels play important roles in the development of a large number of cardiovascular and renal diseases through various molecular mechanisms. In this Special Issue of *Cells*, four original research articles and an up-to-date review present recent advances in our understanding of the complex roles of TRPV4, TRPV1, TRPM4, TRPC6, and TRPA1 in the pathogenesis of cardiovascular and renal diseases.

TRPV4 is abundantly expressed in the distal segments of the renal tubule and is activated in response to mechanical stress, such as shear stress. Pochynyuk et al. previously found that the renal TRPV4 channel is required for adaptation to increased dietary potassium in an aldosterone-dependent manner [1]. In this Special Issue, these authors report their recent discovery that with-no-lysine kinase 1 (WNK1) is essential for the aldosterone-induced up-regulation of TRPV4 expression in distal renal tubules, and the kinase acts by facilitating the translocation of TRPV4 channels to the apical membrane [2]. These findings suggest that TRPV4 might be a downstream or end effector of the aldosterone signaling pathway in the distal renal tubules, through which the channel could play a pivotal role in the maintenance of fluid and electrolyte homeostasis and the regulation of blood pressure.

It is known that aspirin and other salicylates have both beneficial and detrimental effects, including anti-inflammation, sympathetic suppression, and renal impairment. In this Special Issue, Zhong et al. uncovered the complicated relationship between TRPV1 and the effects of salicylates [3]. They found that the sympathetic suppressive effect of salicylates is dependent on TRPV1, while its anti-inflammatory effects are not [3]. Importantly, salicylates-induced renal dysfunction is dependent on intact TRPV1 channels [3], suggesting that salicylates need to be used with caution in treating obesity when the function of TRPV1 channels is impaired.

A number of pathogenic mutations of the *Trpm4* gene have been identified in patients with various forms of cardiac arrhythmias [4]. TRPM4 E7K is a gain-of-function mutation recently identified in patients with progressive conduction blocks and sudden cardiac death [4]. However, the molecular mechanism linking the E7K mutation to functional alterations and clinical phenotypes remains poorly understood. In this Special Issue, Hu et al. revealed that the E7K mutation facilitates TRPM4 channel activation via enhancing its N-terminal interaction with phosphatidylinositol 4,5-bisphosphate (PIP2) [5], which could be one of the pathogenic mechanisms underlying TRPM4 channelopathies.

TRPC6 has been reported to be involved in glomerulosclerosis in patients and in renal fibrosis in mice with ureteral obstruction. Kim and Dryer investigated the role of TRPC6 in age-related renal dysfunction and fibrosis using *Trpc6* gene knockout rats. In this Special Issue, they reported that TRPC6 knockout protected rats from developing glomerulosclerosis but did not improve tubulointerstitial fibrosis or renal dysfunction in 12-month-old, middle-aged rats [6]. These findings suggest that the roles of TRPC6 might be disease model-dependent. However, the role of TRPC6 in renal aging may need to be further investigated in aged, rather than middle-aged, rats.

TRPA1 is unique, as it has a large number of ankyrin-like repeats. This channel is well-known as a regulator of cerebrovascular function. In this Special Issue, Alvarado et al. summarized the evidence on vascular tone control by TRPA1 channels expressed in perivascular nerves and endothelial cells [7]. They also discussed the potential role of TRPA1 in vascular diseases, including hypertension and stroke [7].

Taken together, this Special Issue highlights recent findings regarding the potential roles of several TRP channels in cardiac arrhythmia, obesity-related kidney disease, renal aging, and vascular disease.
